# Impact of Dietetic Counseling Combined With Digital Tools on Obesity‐Related Indices in Greek Adults: The GATEKEEPER Study

**DOI:** 10.1002/oby.70284

**Published:** 2026-09-18

**Authors:** Rafaela Makri, Iliana Evangelou, Eva Karaglani, Maria Vlachava, George E. Dafoulas, Ioanna Drympeta, Konstantinos Votis, Eleni I. Georga, Dimitrios I. Fotiadis, Francisco Lupiáñez‐Villanueva, Frans Folkvord, Leandro Pecchia, Giuseppe Fico, Demosthenes Panagiotakos, Odysseas Androutsos, Yannis Manios, GATEKEEPER study (Greek Use Case 1) group, Odysseas Androutsos, Maria Vlachava, Iliana Evangelou, George E. Dafoulas, Alexandra Bargiota, Alexandra Koubitski, Avgi Karaiskou, Glykeria Papagiannopoulou, Thanasis Topis, Thomas Tzekos, Nelly Mylona, Nikos Papaspanos, Yvonne Katergari, Iphigenia Chionidou, Natasha Davitidou, Vivian Katsarou, Yannis Manios, Eva Karaglani, Rafaela Makri, Ilektra Kalogerakou, Alexandros Karagiannis, Ioanna Nakaki, Elpiniki Vlachopoulou, Maria Dimopoulou, Maria Skaltsa, Ioannis Chrysou, Konstantinos Leontiou, Karamperi Katerina, Evangelia Malakou, Anastasios Papalazarou, Elisavet Parlapani, Anastasia Paschaleri, Christos Paximadas, Maria‐Sofia Pelagidou, Kyriakos Reppas, Katerina Tyrothoulaki, Konstantinos Xenos, Spyridon Zarogiannis, Konstantinos Zervos, Ilias Kalamaras, Dimitrios I. Fotiadis, Eleni I. Georga, Daphne N. Katsarou

**Affiliations:** ^1^ Department of Nutrition and Dietetics, School of Health Science and Education Harokopio University Athens Greece; ^2^ Lab of Clinical Nutrition‐Dietetics, Department of Nutrition‐Dietetics, School of Physical Education, Sport Science and Dietetics University of Thessaly Trikala Greece; ^3^ Faculty of Medicine, School of Health Sciences University of Thessaly Larissa Greece; ^4^ Information Technologies Institute (ITI), Centre for Research and Technology Hellas (CERTH) Thessaloniki Greece; ^5^ Unit of Medical Technology and Intelligent Information Systems, Department of Materials Science and Engineering University of Ioannina Ioannina Greece; ^6^ Foundation for Research and Technology‐Hellas Biomedical Research Institute Ioannina Greece; ^7^ Department of Information and Communication Science Universidad Oberta de Catalunya Barcelona Spain; ^8^ Tilburg School of Humanities and Digital Sciences, Communication and Information Science Tilburg University Tilburg the Netherlands; ^9^ PredictBy Barcelona Spain; ^10^ FacoltÃ Dipartimentale di Ingegneria Università Campus Bio‐Medico Rome Italy; ^11^ ETSIT, Universidad Politécnica de Madrid‐Life Supporting Technologies Research Group Madrid Spain; ^12^ Institute of Agri‐Food and Life Sciences, Hellenic Mediterranean University Research Centre Heraklion Greece; ^13^ European Centre for Obesity Harokopio University Athens Greece

**Keywords:** dietetic counseling, digital tools, GATEKEEPER study, metabolic syndrome, waist circumference

## Abstract

**Objective:**

This study aimed to evaluate the effectiveness of dietetic counseling with digital tools on obesity‐related and metabolic outcomes in adults aged ≥ 55 years at high risk for metabolic syndrome (MetS).

**Methods:**

In this 3‐month randomized controlled trial, 984 adults aged ≥ 55 years with ≥ 1 MetS risk factor were randomized (1:1:1) to: standard care (monthly dietetic counseling), standard care plus web‐based platform, or standard care plus platform and digital devices. Analyses included 954 participants and used linear mixed‐effect models under a missing‐at‐random assumption adjusted for age and sex. The primary outcome was change in waist circumference. Secondary outcomes included BMI, body composition indices, physical activity, and glycemic markers.

**Results:**

Waist circumference decreased in all groups (−6.29, −4.92, −4.69 cm), with smaller reductions in the platform (MD −1.38 cm; 95% CI −2.37 to −0.38) and platform + devices groups (MD −1.61 cm; 95% CI −2.61 to −0.60). BMI, body composition indices, and glycemic markers improved similarly across groups. Physical activity increased more in the platform + devices group (MD +55.52 MET‐min/week; 95% CI 15.32 to 95.72).

**Conclusions:**

Dietetic counseling improved anthropometric and metabolic outcomes in adults at risk for MetS. Digital tools enhanced physical activity but did not provide additional short‐term metabolic benefits.

**Trial Registration:**

ClinicalTrials.gov identifier: NCT05031299

## Study Importance

- Monthly dietetic counseling reduced waist circumference and improved adiposity and metabolic markers in adults ≥ 55 years at risk for MetS.
- Adding a web platform (with or without devices) did not improve short‐term adiposity or glycemic outcomes beyond dietetic counseling alone.
- Wearable/scale support increased physical activity compared with standard care.
- Longer follow‐up is needed to determine whether digital interventions provide sustained cardiometabolic benefits.

## 1 Introduction

Metabolic syndrome (MetS) comprises a cluster of metabolic abnormalities, including central obesity, insulin resistance, atherogenic dyslipidemia, and hypertension. Clinically, MetS is diagnosed when at least three of the following are present: increased waist circumference (WC), elevated fasting plasma glucose, elevated triglycerides, reduced HDL‐cholesterol, and elevated blood pressure. Collectively, these disturbances promote a pro‐inflammatory and pro‐atherogenic state, contributing to endothelial dysfunction and accelerated atherosclerosis [1]. Consequently, MetS is a major contributor to cardiovascular disease (CVD), underlying most coronary heart disease, myocardial infarction, heart failure, hypertension, and stroke events [2, 3, 4].

Central obesity is the key driver of MetS, as dysfunctional visceral adipose tissue promotes insulin resistance, dyslipidemia, and metabolic dysregulation [4, 5]. Addressing metabolic dysfunction is therefore essential for reducing CVD risk [4]. Although advances in prevention and treatment have reduced CVD mortality for decades, rates began rising again in the mid‐2010s, largely due to increasing obesity and related metabolic disorders [6]. Globally, approximately one in four adults is affected by MetS [7]. In Greece, nationally representative prevalence data are limited. Earlier population‐based studies estimated a prevalence of approximately 24% in Greek adults, increasing with age [8]. Substantially higher rates reported in primary care settings in Crete (73.6%) likely reflect older, high‐risk populations [9]. Recent estimates suggest MetS prevalence now exceeds 25%–30% in many adult populations, with the greatest burden among middle‐aged and older adults [10]. Given Greece's high obesity rates and the concurrent shifts away from traditional Mediterranean dietary patterns [11], tailored lifestyle interventions targeting midlife and older adults are urgently needed.

Lifestyle and behavioral modifications remain the first‐line strategy for managing diet‐related noncommunicable diseases such as type 2 diabetes (T2D), prediabetes, hypertension, MetS, and obesity [12, 13]. Although glucagon‐like peptide‐1 receptor agonists (GLP‐1 RAs) have revolutionized the therapeutic landscape of obesity and MetS by demonstrating substantial weight loss, improved glycemic control, and meaningful CVD risk reduction [14], behavioral modifications remain essential for maintaining weight reduction, preserving lean mass, and supporting long‐term weight loss maintenance beyond pharmacotherapy alone [15].

Emerging technologies provide novel opportunities to strengthen lifestyle changes through behavioral modification. Digital health interventions including telehealth, mobile health applications, and wearable devices enhance self‐monitoring of dietary intake, physical activity, and energy expenditure, thereby improving engagement, accountability, and adherence. However, although digital health interventions are increasingly incorporated into chronic disease management, digital literacy in Greece remains below the European Union average, particularly among older adults [16]. Evaluating technology‐supported lifestyle interventions within this demographic context is therefore essential to determine their feasibility and effectiveness in real‐world settings [17].

Adiposity, particularly visceral fat accumulation, strongly predicts CVD risk [18]. While anthropometric indices like body mass index (BMI) and WC are commonly used to assess risk [19], they often neglect lipid and lipoprotein abnormalities linked to dyslipidemia and metabolic dysfunction. Therefore, incorporating multiple adiposity measures provides a more comprehensive assessment of cardiometabolic changes [6].

The present study is part of the GATEKEEPER project Greek Use Case 1 (https://www.gatekeeper‐project.eu/), focused on the development, implementation and evaluation of an intervention combining dietetic counseling with digital tools in adults ≥ 55 years old at high risk for MetS. As the global population ages, the number of adults aged ≥ 55 years is projected to double by 2050. Although advances in diet, lifestyle, education, and healthcare have extended life expectancy, gains in disease‐free years have not kept pace [20]. A previous report from the same randomized controlled trial examined the effects of the intervention on hemodynamic and cardiovascular‐related markers, including blood pressure and vascular parameters [21]. In contrast, the present manuscript focuses on obesity‐related, anthropometric, and glycemic outcomes. We hypothesized that enhanced self‐monitoring and personalized feedback delivered through digital tools would improve adherence to dietary and physical activity recommendations, resulting in greater reductions in central adiposity and improved glycemic control compared to standard care.

## 2 Methods

### 2.1 Ethical Clearance

Participants were informed about the aims and procedures of the Greek GATEKEEPER Greek Use Case 1 and provided written informed consent prior to participation. The study was approved by the Ethics Committees of Harokopio University (No.: C‐2403/12‐10‐2020) and the Department of Nutrition and Dietetics of the University of Thessaly (2/30.11.2020) and was registered at ClinicalTrials.gov (NCT05031299).

### 2.2 Trial Design

Greek Use Case 1 of the GATEKEEPER project was a 3‐month randomized dietary and lifestyle intervention with three study arms: (i) dietetic counseling (standard care group), (ii) dietetic counseling plus a web‐based health‐promotion platform (platform group), and (iii) dietetic counseling plus platform and digital devices (platform + devices group), including an activity tracker (Fitbit Inspire 2) and a digital weight scale (Fitbit Aria Air).

### 2.3 Sample Size and Participants

A priori power analysis indicated that 320 participants per group would provide > 80% power to detect a one standard deviation difference between groups, assuming a 30% attrition. Participants were recruited via local community settings, such as private dietetics practices, Open Care Centers for the Elderly, municipal healthcare centers, and hospitals. Eligibility criteria included: age ≥ 55 years; at least one MetS risk factor (WC > 94 cm [men] or > 80 cm [women], triglycerides ≥ 150 mg/dL, HDL‐C < 40 mg/dL [men] or < 50 mg/dL (women), fasting glucose ≥ 100 mg/dL, or blood pressure ≥ 130/85 mmHg); and independent living. Exclusion criteria included severe hearing or vision impairment, acute or chronic conditions limiting participation, dementia or cognitive impairment, institutionalization, or participation in another trial.

A total of 1200 individuals were screened, and 984 participants (26% men, 74% women) were enrolled. Participants were distributed between the Attica region and Central Greece in a 70:30 ratio, reflecting population distribution reported in the 2021 national census data.

### 2.4 Randomization

Participants were randomly assigned (1:1:1) using a computer‐generated sequence developed in MS Excel by independent personnel. Randomization was not stratified or blocked; therefore, minor imbalances in group sizes and baseline characteristics are attributable to chance. Of the 984 randomized participants, 344 were allocated to standard care, 320 to the platform group, and 320 to the platform + devices group. After excluding 30 participants with incomplete baseline data, the final analysis included 954 participants (340 standard care, 313 platform, 301 platform + devices).

**FIGURE 1:**
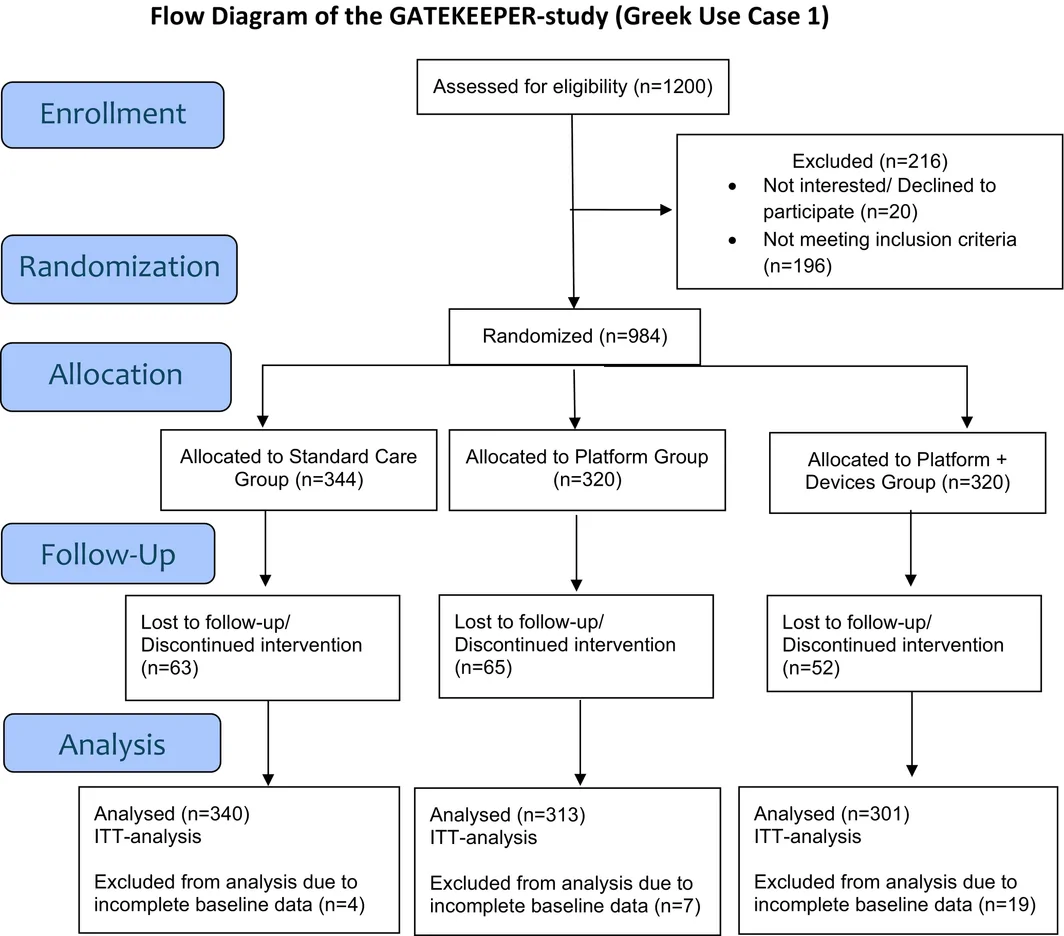
GATEKEEPER project Greek Use Case 1 flowchart. Allocation, follow‐up, and analysis. Lost to follow‐up: If the participant missed the scheduled contact time points and attempts at communicating with the patient were unsuccessful, the participant was considered lost to follow‐up. ITT‐analysis, intention‐to‐treat analysis. [Color figure can be viewed at wileyonlinelibrary.com]

A CONSORT flow diagram is presented in Figure 1.

### 2.5 Interventions

All participants received monthly face‐to‐face dietetic counseling focused on dietary assessment, nutrition education, lifestyle modification, and individualized diet plans. Specifically:

- **Standard care group:** Received dietetic counseling according to national healthcare guidelines for individuals with MetS risk factors.
- **Platform group:** Received standard care plus access to a web‐based health‐promotion platform for self‐monitoring, goal setting, appointment scheduling, meal planning, and communication with dietitians.
- **Platform + devices group:** Received standard care, platform access, and additional digital devices, a digital weighing scale and a smartwatch or wristband for monitoring physical activity and sleep patterns. Device data were synchronized with the platform.

The health‐promotion platform, developed by the Centre for Research and Technology Hellas (CERTH), supported self‐management and behavioral monitoring. Participants could log dietary intake, track physiological metrics (e.g., heart rate zones), and receive personalized recommendations. Device data were automatically synchronized, with optional manual entry of additional health information. Dietitians monitored adherence, adjusted plans, and provided personalized reminders, feedback, and motivational messages through the platform.

The intervention lasted 3 months (±1 week) and followed a pre–post design. It was implemented in Attica and Central Greece (10 cities: Trikala, Karditsa, Larissa, Volos, Lamia, Grevena, Katerini, Veria, Ioannina, and Kozani) by 40 dietitians (30 in Attica, 10 in Central Greece).

### 2.6 Outcomes

Assessments were performed at baseline and after 3 months (follow‐up). The primary outcome was change in WC. Secondary outcomes included BMI, body fat percentage, visceral fat index, muscle mass, fasting glucose, HbA1c, and physical activity.

### 2.7 Anthropometric Measurements

WC was measured as an indicator of central adiposity and visceral fat accumulation, recorded in centimeters (cm). Participants wore light clothing and removed belts or accessories. Measurements were taken in a relaxed standing position at the natural waist between the lower rib and iliac crest, using a nonstretchable tape available at the participating centers. The tape was positioned horizontally and kept parallel to the floor. Measurements were recorded at the end of a normal exhalation, with the abdomen relaxed, and the mean of three measurements was used.

Body weight was measured using a calibrated digital scale with participants wearing light clothing and no shoes. Height was measured with a stadiometer to the nearest centimeter. Participants were instructed to stand straight with their back against the stadiometer, with their feet together. BMI was calculated as weight (kg) divided by height squared (m^2^) and classified according to World Health Organization (WHO) criteria [22].

### 2.8 Body Composition Analysis

Body composition analysis was assessed via bioelectrical impedance analysis (BIA). Participants were instructed to avoid large meals or alcohol for 4 h and strenuous activity for 12 h before measurement to minimize hydration‐related variability. Assessments were performed using a calibrated BIA device at each participating center, under standardized conditions. Measured parameters included body fat percentage (%), muscle mass (kg), and visceral fat index. These reflect adiposity, lean tissue mass, and abdominal fat accumulation, respectively.

### 2.9 Biochemical Markers

Fasting blood samples were collected at baseline and follow‐up after an overnight fast of 8–12 h. Fasting plasma glucose (mg/dL) was measured using enzymatic methods, and glycated hemoglobin (HbA1c, %) was assessed using high‐performance liquid chromatography (HPLC) or equivalent standardized assays aligned with international reference standards. HbA1c reflects average glycemic control over the preceding 2–3 months.

### 2.10 Physical Activity

Physical activity was assessed using the International Physical Activity Questionnaire–Short Form (IPAQ‐SF). The Greek version of the IPAQ‐SF has been translated and validated in adult populations, demonstrating acceptable reliability and validity for assessing physical activity in Greek adults [23, 24, 25]. This seven‐item questionnaire captures time spent in walking, moderate and vigorous activity, and sedentary behavior during the previous 7 days. Moderate activity was defined as exercise causing a slight increase in breathing or heart rate (e.g., brisk walking, casual cycling, dancing), while vigorous activity required substantial effort and heavy breathing (e.g., running, lap swimming, basketball, cycling uphill, tennis, HIIT). Participants reported activity across all domains. Activity levels were expressed as metabolic equivalent minutes per week (MET‐min/week) calculated as:

- **Low** = 3.3 × [walking minutes] × [walking days].
- **Moderate** = 4.0 × [moderate‐intensity minutes] × [moderate days].
- **Vigorous** = 8.0 × [vigorous‐intensity minutes] × [vigorous‐intensity days] [26].

Total physical activity score was the sum of all intensities. Sitting time was recorded but excluded from total scores.

### 2.11 Statistical Analysis

Baseline characteristics were summarized using means and standard deviations (SD) for continuous variables, frequencies and percentages for categorical variables, and medians with interquartile ranges (IQR) for skewed data. Baseline group balance was assessed using standardized mean differences (SMD), with values > 0.20 indicating potential imbalance.

Changes in outcomes from baseline to follow‐up were analyzed using linear mixed‐effects models. Fixed effects included group, time (baseline and 3‐month follow‐up), and their interaction, with random intercepts to account for within‐subject correlation over time. Models were adjusted for age and sex.

Intervention effects were evaluated using group‐by‐time interaction and reported as adjusted mean differences with 95% confidence intervals (CI). Between‐group differences in change (ΔΔ) were calculated as the adjusted change in the standard care group minus the adjusted change in each intervention group. Negative ΔΔ values indicate greater reductions in the standard care group, whereas positive values indicate greater reductions in the intervention group. Visceral fat index was analyzed similarly, despite being presented as median values at baseline.

Sensitivity analyses were additionally adjusted for baseline physical activity score and education level. Analyses followed intention‐to‐treat (ITT) principle, excluding participants with missing baseline data, while participants with incomplete follow‐up were retained under a missing‐at‐random assumption. Statistical analyses were performed using IBM SPSS Statistics, version 21.0, with 95% confidence intervals assessed at the 95% confidence level.

## 3 Results

### 3.1 Baseline Characteristics

Baseline characteristics by intervention group are presented in Table 1. Mean age was higher in the standard care group (64.4 ± 7.0 years), compared with the Platform (61.9 ± 5.7 years) and Platform + Devices groups (61.1 ± 5.7 years), indicating a moderate imbalance (SMD > 0.20). Sex distribution was generally comparable, although women predominated across all groups (77.1% in standard care, 68.4% in Platform, and 76.4% in Platform + Devices). Region of residence, marital status, sedentary time, and WC were well balanced across groups. Participants in the intervention groups had a higher proportion of individuals with more than 12 years of education and slightly higher physical activity scores compared to the standard care group (SMD up to 0.30). Anthropometric and metabolic measures, including BMI, fasting blood glucose, and HbA1c, were similar across groups. Body fat percentage was higher in the intervention groups (SMD > 0.20), while visceral fat index (median, IQR) was comparable. Overall, baseline differences were small to moderate.

**TABLE 1:** Baseline characteristics of the study participants (*n* = 954) between groups.

	Standard Care group (*n* = 340)	Platform group (*n* = 313)	Platform + Devices group (*n* = 301)	SMD^1^	SMD^2^
Age, years	64.4 ± 7.0	61.9 ± 5.7	61.1 ± 5.7	**0.39**	**0.52**
Sex, *n* (%)					
Male	78 (22.9)	99 (31.6)	71 (23.6)	**0.20**	0.02
Female	262 (77.1)	214 (68.4)	230 (76.4)		
Region, *n* (%)					
Urban	224 (65.9)	213 (68.1)	221 (73.4)	0.05	0.16
Rural	116 (34.1)	100 (31.9)	80 (26.6)		
Education level, *n* (%)					
≤ 12 years	151 (51.5)	114 (38.3)	104 (36.6)	**0.27**	**0.30**
> 12 years	142 (48.5)	184 (61.7)	180 (63.4)		
Marital status, *n* (%)					
Married/cohabiting	212 (69.7)	215 (71.4)	212 (74.4)	0.04	0.10
Single/widowed/divorced	92 (30.3)	86 (28.6)	73 (25.6)		
Lifestyle					
Total sedentary time, h per day	6.4 ± 3.5	6.7 ± 3.4	6.6 ± 3.3	0.08	0.04
Physical activity score (IPAQ‐SF)	275.1 ± 174.2	320.7 ± 221.5	333.5 ± 214.6	**0.23**	**0.30**
Anthropometric measurements					
Weight, kg	88.7 ± 17.3	91.2 ± 20.4	89.2 ± 17.3	*	*
Height, m	1.63 ± 0.1	1.65 ± 0.1	1.65 ± 0.1	*	*
BMI, kg/m^2^	33.5 ± 6.1	33.3 ± 6.0	32.8 ± 5.7	0.03	0.13
Waist circumference, cm	108.1 ± 13.5	110.1 ± 16.2	107.4 ± 13.5	0.00	0.03
Body fat percentage, %	40.7 ± 8.0	38.7 ± 6.9	38.7 ± 7.3	**0.26**	**0.26**
Body muscle mass, kg	49.5 ± 11.3	51.6 ± 14.2	51.5 ± 12.0	0.16	0.17
Visceral fat, points	13.0 (11.0–15.0)	13.0 (10.0–17.0)	12.0 (10.0–15.0)		
Glycemic status					
Fasting blood glucose, mg/dL	107.9 ± 21.4	107.9 ± 23.6	104.7 ± 17.6	0.00	0.27
HbA1c, %	6.1 ± 1.0	6.0 ± 1.0	5.8 ± 0.7	0.06	**0.32**

*Note:* Values are means ± SD or percentages (%) or medians (25th–75th percentile). SMD = standard mean difference; SMD^1^ = Platform group vs. standard care group; SMD^2^ = Platform + devices vs. standard care group. * = SMD was not calculated for height and weight as these variables were reflected in BMI. Bold values indicate an absolute SMD ≥ 0.20, suggesting a meaningful baseline imbalance between groups.

### 3.2 Changes From Baseline to 3 Months

Adjusted mean changes in anthropometric, lifestyle, and glycemic outcomes are presented in Tables 2 and 3.

**TABLE 2:** Adjusted means by group from baseline to 3 months and within‐group comparisons.

Outcome	Allocated study group	Baseline mean (95% CI)	3‐month follow‐up, mean (95% CI)	Difference in change (3 months–baseline)
Anthropometrics				
Waist circumference, cm	Standard Care	110.80 (108.91 to 112.70)	104.51 (102.58 to 106.44)	−6.29 (−7.04 to −5.55)
	Platform	112.08 (110.40 to 113.77)	107.16 (105.45 to 108.88)	−4.92 (−5.93 to −3.92)
	Platform + Devices	110.22 (108.41 to 112.02)	105.53 (103.70 to 107.36)	−4.69 (−5.69 to −3.68)
BMI, kg/m^2^	Standard Care	33.67 (32.99 to 34.35)	32.01 (31.33 to 32.69)	−1.66 (−1.83 to −1.49)
	Platform	33.37 (32.70 to 34.04)	32.13 (31.45 to 32.80)	−1.24 (−1.42 to −1.07)
	Platform + Devices	32.78 (32.07 to 33.50)	31.33 (30.62 to 32.04)	−1.45 (−1.63 to −1.28)
Body fat, %	Standard Care	37.80 (37.02 to 38.58)	35.44 (34.65 to 36.28)	−2.36 (−2.76 to −1.95)
	Platform	36.79 (36.06 to 37.53)	34.66 (33.90 to 35.42)	−2.14 (−2.56 to −1.72)
	Platform + Devices	36.05 (35.27 to 36.84)	33.65 (32.85 to 34.46)	−2.40 (−2.83 to −1.98)
Body muscle mass, kg	Standard Care	54.64 (53.34 to 55.94)	54.33 (53.03 to 55.63)	−0.31 (−0.63 to 0.02)
	Platform	54.47 (53.27 to 55.67)	54.29 (53.07 to 55.49)	−0.19 (−0.51 to 0.14)
	Platform + Devices	55.74 (54.48 to 57.01)	55.32 (54.04 to 56.59)	−0.43 (−0.75 to −0.11)
Visceral fat	Standard Care	14.60 (14.03 to 15.18)	13.52 (12.94 to 14.10)	−1.08 (−1.28 to −0.88)
	Platform	15.28 (14.79 to 15.77)	14.55 (14.05 to 15.04)	−0.74 (−0.92 to −0.55)
	Platform + Devices	14.86 (14.34 to 15.38)	13.82 (13.30 to 14.34)	−1.04 (−1.21 to −0.87)
Lifestyle				
Physical activity score	Standard Care	276.58 (251.18 to 301.98)	315.69 (286.02 to 345.35)	39.10 (10.40 to 67.81)
	Platform	321.14 (296.08 to 346.20)	373.10 (344.24 to 401.97)	51.96 (23.34 to 80.59)
	Platform + Devices	333.92 (307.86 to 359.99)	428.55 (399.52 to 457.57)	94.62 (66.49 to 122.76)
Sedentary behavior, h	Standard Care	6.26 (5.88 to 6.63)	5.62 (5.20 to 6.04)	−0.64 (−1.03 to −0.24)
	Platform	6.70 (6.33 to 7.07)	5.72 (5.29 to 6.15)	−0.98 (−1.41 to −0.56)
	Platform + Devices	6.59 (6.20 to 6.98)	5.79 (5.36 to 6.22)	−0.80 (−1.21 to −0.39)
Glycemic status				
Fasting blood glucose, mg/dL	Standard Care	109.82 (106.85 to 112.79)	101.61 (97.89 to 105.33)	−8.21 (−11.31 to −5.11)
	Platform	109.30 (106.64 to 111.97)	102.21 (98.85 to 105.56)	−7.09 (−9.99 to −4.20)
	Platform + Devices	106.76 (104.04 to 109.48)	101.68 (98.48 to 104.88)	−5.08 (−7.70 to −2.47)
HbA1c, %	Standard Care	6.11 (5.97 to 6.26)	5.82 (5.65 to 5.99)	−0.29 (−0.41 to −0.17)
	Platform	6.04 (5.90 to 6.18)	5.82 (5.67 to 5.97)	−0.22 (−0.32 to −0.11)
	Platform + Devices	5.87 (5.73 to 6.01)	5.70 (5.55 to 5.84)	−0.18 (−0.27 to −0.09)

*Note:* Values are estimated marginal means (95% CI) from linear mixed‐effects models adjusted for age and sex.

**TABLE 3:** Between‐group differences in change from baseline to 3 months.

Outcome	Comparison	Difference in change	95% CI
Anthropometrics			
Waist circumference, cm	Platform vs. Standard Care	−1.38	**−2.37 to −0.38**
	Platform + Devices vs. Standard Care	−1.61	**−2.61 to −0.60**
BMI, kg/m^2^	Platform vs. Standard Care	−0.42	**−0.66 to −0.18**
	Platform + Devices vs. Standard Care	−0.21	−0.45 to 0.04
Body fat, %	Platform vs. Standard Care	−0.22	−0.81 to 0.37
	Platform + Devices vs. Standard Care	0.05	−0.54 to 0.64
Body muscle mass, kg	Platform vs. Standard Care	−0.12	−0.58 to 0.33
	Platform + Devices vs. Standard Care	0.12	−0.34 to 0.57
Visceral fat	Platform vs. Standard Care	−0.34	**−0.61 to −0.07**
	Platform + Devices vs. Standard Care	−0.04	−0.31 to 0.23
Lifestyle			
Physical activity score	Platform vs. Standard Care	12.86	**−27.68 to 53.40**
	Platform + Devices vs. Standard Care	55.52	**15.32 to 95.72**
Sedentary behavior, h	Platform vs. Standard Care	0.35	−0.23 to 0.92
	Platform + Devices vs. Standard Care	0.16	−0.41 to 0.73
Glycemic status			
Fasting blood glucose, mg/dL	Platform vs. Standard Care	−1.12	−5.36 to 3.12
	Platform + Devices vs. Standard Care	−3.13	−7.19 to 0.93
HbA1c, %	Platform vs. Standard Care	−0.07	−0.23 to 0.09
	Platform + Devices vs. Standard Care	−0.11	−0.26 to 0.03

*Note:* Differences in change represent adjusted between‐group differences (ΔΔ), calculated as Standard Care minus intervention (Platform or Platform + Devices group), derived from mixed‐effects models. Negative values indicate greater reductions in the Standard Care group, whereas positive values indicate greater reductions in the intervention group. Bold values indicate statistically significant between‐group differences (95% CI does not include 0).

### 3.3 Obesity‐Related Indices and Body Composition Outcomes

WC, the primary outcome, decreased in all groups over 3 months (Table 2). The standard care group showed a mean reduction of 6.29 cm (95% CI 5.55 to 7.04), while the Platform and the Platform + Devices groups showed mean reductions of 4.92 cm (95% CI 3.92 to 5.93) and 4.69 cm (95% CI 3.68 to 5.69), respectively. Although reductions were observed across all groups, group‐by‐time interaction indicated smaller reductions in both intervention groups compared with the standard care group, with adjusted between‐group differences of −1.38 cm (95% CI −2.37 to −0.38) for the Platform group and −1.61 cm (95% CI −2.61 to −0.60) for the Platform + Devices group (Table 3).

BMI decreased across all groups, with within‐group reductions ranging from −1.24 to −1.66 kg/m^2^ (Table 2). Between‐group comparisons showed a greater reduction in BMI in the standard care group compared to the Platform group, while no between‐group difference was observed for the Platform + Devices group (Table 3). Body fat percentage decreased across all groups over the 3‐month period, with comparable reductions across study groups. The standard care group experienced a mean reduction of 2.36% (95% CI −2.76% to −1.95%), while Platform group and Platform + Devices group showed mean reductions of 2.14% (95% CI −2.53% to −1.75%) and 2.40% (95% CI −2.81% to −1.99%), respectively. Differences in the extent of body fat percentage reduction between the intervention groups and the control group were minimal (Table 3). Visceral fat index decreased from baseline to 3 months in all groups. Between‐group comparisons indicated a greater reduction in visceral fat in the standard care group compared with the Platform group (−0.34, 95% CI −0.61 to −0.07), while no between‐group difference was observed for the Platform + Devices group (−0.04, 95% CI −0.31 to 0.23) (Table 3). Body muscle mass remained relatively stable from baseline to the 3‐month follow‐up across all groups. Mean changes were small across groups, ranging from −0.19 kg in the Platform group, to −0.31 kg in the standard care group, to −0.43 kg in the Platform + Devices group (Table 2). Adjusted between‐group comparisons did not demonstrate differences in muscle mass change between the intervention groups and the standard care group (Table 3).

### 3.4 Lifestyle Outcomes

Physical activity score increased across all groups over the 3‐month period (Table 2). The standard care group showed a mean increase of 39.10 units (95% CI 10.40 to 67.81), while increases of 51.96 units (95% CI 23.34 to 80.59) and 94.62 units (95% CI 66.49 to 122.76) were observed in the Platform group and the Platform + Devices group, respectively. Between‐group comparisons indicated a larger increase in physical activity score in the Platform + Devices group compared to the standard care group (55.52, 95% CI 15.32 to 95.72), whereas no between‐group difference was observed for the Platform group (12.86, 95% CI −27.68 to 53.40) (Table 3). Sedentary time decreased across all groups from baseline to 3‐month follow‐up, with mean reductions ranging from 0.64 to 0.98 h per day (Table 2). Between‐group comparisons did not indicate differences in changes in sedentary behavior between the intervention groups and the standard care group (Table 3).

### 3.5 Glycemic Status

Fasting blood glucose levels decreased across from baseline to 3‐month follow‐up (Table 2). The standard care group showed a mean reduction of 8.21 mg/dL (95% CI −11.31 to −5.11), while reductions of 7.09 mg/dL (95% CI −9.99 to −4.20) and 5.08 mg/dL (95% CI −7.70 to −2.47) were observed in the Platform group and the Platform + Devices group, respectively. Between‐group comparisons did not indicate differences in fasting blood glucose change between the intervention groups and the standard care group. HbA1c levels also declined in all groups from baseline to 3‐month follow‐up (Table 2). Mean reductions were 0.29% (95% CI −0.41% to −0.17%) in the standard care group, 0.22% (95% CI −0.32% to −0.11%) in the Platform group, and 0.18% (95% CI −0.27% to −0.09%) in the Platform + Devices group. No between‐group differences were observed in changes in HbA1c (Table 3).

Results from sensitivity analyses additionally adjusting for baseline physical activity score and education level were consistent with the primary analyses (data not shown).

## 4 Discussion

In Greece, MetS is highly prevalent and increases with age, although estimates vary depending on population and diagnostic criteria [8, 9]. These trends highlight the urgent need for effective lifestyle and behavioral interventions to promote healthy aging and physical activity [13, 21, 27, 28]. Within the framework of the GATEKEEPER project, Greek Use Case 1 evaluated the effects of nutritional counseling combined with digital tools in adults aged ≥ 55 years at high risk for MetS. In this randomized controlled trial, a 3‐month dietetic counseling intervention resulted in significant improvements in obesity‐related and metabolic outcomes across all study groups. WC, BMI, body fat percentage, visceral fat index, fasting glucose, and HbA1c decreased significantly from baseline, highlighting the effectiveness of structured counseling in reducing cardiometabolic risk. These findings complement those of a previous report from the same trial, which demonstrated beneficial effects of the intervention on hemodynamic and cardiovascular‐related markers [20]. Together, these results suggest that nutritional counseling, either alone or combined with digital tools, can improve multiple dimensions of cardiometabolic health in adults ≥ 55 years at high risk for MetS [21].

Contrary to our initial hypothesis, the addition of a web‐based health platform, with or without wearable devices, did not result in greater reductions in WC or other anthropometric measures compared to standard care. In fact, greater reductions in WC and visceral fat were observed in the standard care group compared with the platform group. These findings suggest that monthly face‐to‐face dietetic counseling alone may be sufficient to achieve meaningful short‐term improvements in adiposity among midlife and older adults. Our findings do not support the hypothesis that the addition of digital tools confers superior short‐term improvements in obesity‐related outcomes compared to dietetic counseling alone, in contrast with findings from other studies. This discrepancy may be explained by variability in participant engagement, the short follow‐up period, or the possibility that the technological components, while supportive, were not intensive or interactive enough to promote additional weight loss beyond dietetic counseling.

Nevertheless, important differences emerged in lifestyle outcomes. Participants in the platform + devices group demonstrated significantly greater increases in physical activity compared to standard care, supporting evidence that wearable devices enhance self‐monitoring and promote behavioral engagement [29]. Previous meta‐analyses have shown that digital and app‐based interventions can produce modest but significant improvements in weight and cardiometabolic outcomes, particularly when incorporating behavior change techniques such as goal setting and real‐time feedback [30]. However, effect sizes are generally small, with greater effectiveness observed when combined with structured professional support.

Several factors may explain the absence of superior anthropometric outcomes in the digital intervention arms. First, baseline digital literacy levels among older Greek adults may have influenced engagement with the platform. Second, adherence to digital self‐monitoring may decline over time without intensive reinforcement, and third, the relatively short duration of the intervention (3 months) may have limited the capacity to detect incremental benefits of digital augmentation beyond structured counseling.

Muscle mass remained stable across groups despite weight and fat reductions, suggesting that the intervention preserved lean tissue, a clinical consideration in older populations. Furthermore, improvements in fasting blood glucose and HbA1c across all groups underscore the metabolic relevance of dietary counseling, even in the absence of additional digital support.

The strengths of this study include its large sample size, randomized design, real‐world implementation in specific age‐group population across multiple regions, and comprehensive assessment of anthropometric and metabolic outcomes. However, several limitations should be acknowledged. The intervention was relatively short, BIA‐derived body composition measures provided indirect estimates compared with gold‐standard techniques, and different BIA devices were used influencing the accuracy of the method. This introduces the possibility of measurement bias, highlighting the importance of consistent equipment in future studies. In addition, researcher‐related bias cannot be entirely excluded in anthropometric assessments such as height and WC. Importantly, no formal process evaluation of the digital tools was conducted. Differential engagement with digital tools and devices was not systematically quantified, and adherence, usability, and implementation fidelity were not formally assessed. As a result, the extent to which the participants actively interacted with the digital components remains unclear, limiting the interpretation of their true impact. As participants were mostly older adults, sustaining participant engagement throughout the study proved challenging. Limited technological literacy and concerns regarding data privacy may have reduced intervention effectiveness. Although the platform incorporated a user‐friendly interface, training sessions, and GDPR‐compliant privacy safeguards to enhance trust and usability, additional technical support and structured engagement strategies may be required in future implementations.

## 5 Conclusion

In this randomized controlled trial of adults aged ≥ 55 years at high risk of MetS, structured dietetic counseling significantly improved obesity‐related and glycemic outcomes over a 3‐month period. The addition of a digital health platform, with or without wearable devices, did not confer additional short‐term benefits in anthropometric or metabolic parameters beyond standard care, although the combined platform + devices intervention significantly enhanced physical activity levels. These findings suggest that while digital tools may strengthen behavioral engagement, particularly for physical activity, structured dietetic counseling remains a cornerstone of effective cardiometabolic risk reduction in midlife and older adults. Future studies should explore longer term follow‐up strategies to optimize digital engagement to maximize clinical impact. Addressing challenges related to digital literacy and sustained user engagement is critical to ensuring that technological solutions are inclusive, effective, and accessible to all target populations. These findings support the growing body of evidence that structured dietetic counseling can improve obesity‐related and metabolic outcomes in midlife and older adults at risk of MetS. While digital tools and wearable devices may enhance behavioral engagement, particularly physical activity, they did not confer additional short‐term benefits in anthropometric or metabolic outcomes beyond standard care. Future studies should investigate longer‐term follow‐up and strategies to optimize sustained digital engagement and maximize the clinical impact of personalized digital support.

## Author Contributions

**Rafaela Makri:** writing – original draft (lead), investigation (equal), formal analysis (equal), writing – review and editing (equal). **Iliana Evangelou:** investigation (equal), writing – review and editing (equal). **Eva Karaglani:** conceptualization (supporting), methodology (supporting), writing – review and editing (equal). **Maria Vlachava:** conceptualization (supporting), methodology (supporting), writing – review and editing (equal). **George E. Dafoulas:** funding acquisition (equal), methodology (supporting), writing – review and editing (equal). **Ioanna Drympeta:** writing – review and editing (equal). **Konstantinos Votis:** writing – review and editing (equal). **Eleni I. Georga:** writing – review and editing (equal). **Dimitrios I. Fotiadis:** project administration (equal), writing – review and editing (equal). **Francisco Lupiáñez‐Villanueva:** project administration (supporting), writing – review and editing (equal). **Frans Folkvord:** project administration (supporting), writing – review and editing (equal). **Leandro Pecchia:** project administration (lead), writing – review and editing (equal). **Giuseppe Fico:** project administration (equal), writing – review and editing (equal). **Demosthenes Panagiotakos:** formal analysis (equal), writing – review and editing (equal). **Odysseas Androutsos:** conceptualization (lead), data curation (equal), funding acquisition (equal), methodology (lead), supervision (equal), writing – review and editing (equal). **Yannis Manios:** conceptualization (lead), data curation (equal), funding acquisition (equal), methodology (lead), supervision (equal), writing – review and editing (equal).

## Funding

This project was supported by the European Union's Horizon 2020 research and innovation programme under grant agreement No. 857223.

## Conflicts of Interest

The authors declare no conflicts of interest.

## Data Availability

The data presented in this study are available on request from the corresponding author. The data are not publicly available due to data protection restrictions.

## Appendix A GATEKEEPER Study Greek Use Case 1 Group

*University of Thessaly (Greece)*: Odysseas Androutsos, Maria Vlachava, Iliana Evangelou, George E. Dafoulas, Alexandra Bargiota, Alexandra Koubitski, Avgi Karaiskou, Glykeria Papagiannopoulou, Thanasis Topis, Thomas Tzekos, Nelly Mylona, Nikos Papaspanos, Yvonne Katergari, Iphigenia Chionidou, Natasha Davitidou, Vivian Katsarou. *Harokopio University (Greece)*: Yannis Manios, Eva Karaglani, Rafaela Makri, Ilektra Kalogerakou, Alexandros Karagiannis, Ioanna Nakaki, Elpiniki Vlachopoulou, Maria Dimopoulou, Maria Skaltsa, Ioannis Chrysou, Konstantinos Leontiou, Karamperi Katerina, Evangelia Malakou, Anastasios Papalazarou, Elisavet Parlapani, Anastasia Paschaleri, Christos Paximadas, Maria‐Sofia Pelagidou, Kyriakos Reppas, Katerina Tyrothoulaki, Konstantinos Xenos, Spyridon Zarogiannis, Konstantinos Zervos. *Centre for Research and Technology Hellas (CERTH) (Greece)*: Ilias Kalamaras. *University of Ioannina (UOI) (Greece)*: Dimitrios I. Fotiadis, Eleni I. Georga, Daphne N. Katsarou.
